# Production of MHCII‐expressing classical monocytes increases during aging in mice and humans

**DOI:** 10.1111/acel.13701

**Published:** 2022-08-30

**Authors:** Pijus K. Barman, Juliana E. Shin, Sloan A. Lewis, Seokjo Kang, Di Wu, Yizhou Wang, Xiaoming Yang, Prakash S. Nagarkatti, Mitzi Nagarkatti, Ilhem Messaoudi, Bérénice A. Benayoun, Helen S. Goodridge

**Affiliations:** ^1^ Board of Governors Regenerative Medicine Institute Cedars‐Sinai Medical Center Los Angeles California USA; ^2^ Research Division of Immunology in the Department of Biomedical Sciences Cedars‐Sinai Medical Center Los Angeles California USA; ^3^ Department of Molecular Biology and Biochemistry University of California Irvine California USA; ^4^ Institute for Immunology University of California Irvine California USA; ^5^ Applied Genomics, Computation and Translational Core, Cedars‐Sinai Cancer Cedars‐Sinai Medical Center Los Angeles California USA; ^6^ Department of Pathology, Microbiology and Immunology, School of Medicine University of South Carolina Columbia South Carolina USA; ^7^ Department of Microbiology, Immunology and Molecular Genetics in the College of Medicine University of Kentucky Lexington Kentucky USA; ^8^ Leonard Davis School of Gerontology University of Southern California Los Angeles California USA; ^9^ Molecular and Computational Biology Department, USC Dornsife College of Letters, Arts and Sciences University of Southern California Los Angeles California USA; ^10^ Biochemistry and Molecular Medicine Department, USC Keck School of Medicine University of Southern California Los Angeles California USA

**Keywords:** aging, bone marrow progenitors, monocytes

## Abstract

Aging is associated with increased monocyte production and altered monocyte function. Classical monocytes are heterogenous and a shift in their subset composition may underlie some of their apparent functional changes during aging. We have previously shown that mouse granulocyte‐monocyte progenitors (GMPs) produce “neutrophil‐like” monocytes (NeuMo), whereas monocyte‐dendritic cell progenitors (MDPs) produce monocyte‐derived dendritic cell (moDC)‐producing monocytes (DCMo). Here, we demonstrate that classical monocytes from the bone marrow of old male and female mice have higher expression of DCMo signature genes (*H2‐Aa*, *H2‐Ab1*, *H2‐Eb1*, *Cd74*), and that more classical monocytes express MHCII and CD74 protein. Moreover, we show that bone marrow MDPs and classical monocytes from old mice yield more moDC. We also demonstrate higher expression of *Aw112010* in old monocytes and that *Aw112010* lncRNA activity regulates MHCII induction in macrophages, which suggests that elevated *Aw112010* levels may underlie increased MHCII expression during monocyte aging. Finally, we show that classical monocyte expression of MHCII is also elevated during healthy aging in humans. Thus, aging‐associated changes in monocyte production may underlie altered monocyte function and have implications for aging‐associated disorders.

## Graphical abstract

During aging, monocyte‐dendritic cell progenitors (MDPs) in bone marrow produce more MHCII‐expressing classical monocytes (DCMo) and more monocyte‐derived DCs (moDC). Classical monocyte expression of the lncRNA *AW112010* also increases during aging in mice and *AW112010* promotes MHCII expression.

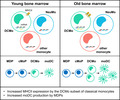

**Abbreviations**
cMoP: MDP‐derived monocyte‐committed progenitor
DCMo: dendritic cell‐producing monocyte
DEG: differentially expressed gene
GMP: granulocyte‐monocyte progenitor
lncRNA: long non‐coding RNA
MDP: monocyte‐dendritic cell progenitor
moDC: monocyte‐derived dendritic cell
MP: GMP‐derived monocyte‐committed progenitor
NeuMo: neutrophil‐like monocyte
scRNAseq: single‐cell RNA sequencing

## 1 INTRODUCTION

Monocytes are innate immune cells with a variety of roles in anti‐microbial defense, tissue repair, and antigen presentation. Three major subtypes of monocytes have been documented in the steady‐state and during infection and injury in humans and mice: (i) “classical” monocytes (Ly6C^hi^ in mice and CD14^+^ CD16^−^ in humans), which are recruited to inflamed tissues, (ii) “non‐classical” monocytes (Ly6C^lo^ in mice and CD14^lo^ CD16^+^ in humans), which patrol and repair the vascular endothelium, and (iii) “intermediate” monocytes (Ly6C^int^ in mice and CD14^+^ CD16^+^ in humans), which are thought to represent a transition state between classical and non‐classical monocytes (Auffray et al., 2007; Carlin et al., 2013; Geissmann et al., 2003; Shi & Pamer, 2011). Recently, multiparametric single‐cell studies have revealed further heterogeneity among monocytes within the classical and non‐classical subsets (Guilliams et al., 2018; Trzebanski & Jung, 2020; Wolf et al., 2019). We previously demonstrated that monocytes are produced independently by granulocyte‐monocyte progenitors (GMPs) and monocyte‐dendritic cell progenitors (MDPs) in mouse bone marrow (Yáñez et al., 2017), and subsequent studies confirmed our observation that the GMP pathway gives rise to neutrophil‐like monocytes (NeuMo) whereas the MDP pathway yields monocyte‐derived dendritic cell (moDC)‐producing monocytes (DCMo) (Tusi et al., 2018; Weinreb et al., 2020; Yáñez et al., 2017).

Aging is characterized by chronic low‐level inflammation (inflammaging) and a progressive decline in immune cell function (immunosenescence), which lead to attenuated host responses against infections and vaccines, as well as defective tissue repair (Aiello et al., 2019; Gruver et al., 2007). Aging increases the number of both classical and non‐classical monocytes in humans and mice due to myeloid‐biased hematopoiesis (Dykstra et al., 2011; Grover et al., 2016; Ho et al., 2019; Puchta et al., 2016; Seidler et al., 2010). Monocyte functions are also altered during aging. For instance, increased production of pro‐inflammatory cytokines such as TNF‐α (Hearps et al., 2012), decreased mitochondrial respiration (Pence & Yarbro, 2018) and faulty lipid metabolism (Saare et al., 2020) have been reported in humans, and mouse studies have revealed impaired pathogen clearance by monocytes due to reduced pathogen binding, and defective phagocytosis of senescent neutrophils by aged macrophages (Frisch et al., 2019; Puchta et al., 2016; Wong et al., 2017). The aging‐associated risk of atherosclerosis has also been attributed to increased recruitment of monocytes into atherosclerotic lesions (Tyrrell & Goldstein, 2021).

Recent transcriptomic and epigenomic studies have highlighted decreased expression of genes associated with energy metabolism and protein synthesis (Reynolds et al., 2015; Saare et al., 2020) and differential methylation of histones and DNA (Cheung et al., 2018; Reynolds et al., 2014; Shchukina et al., 2021) in peripheral blood monocytes from older individuals. In addition, aging‐associated changes in the transcriptome and epigenome of human peripheral blood mononuclear cells (PBMCs) were shown to differ between males and females, indicating that sexual dimorphism impacts aging‐related changes in monocytes (Gal‐Oz et al., 2019; Márquez et al., 2020; So et al., 2021). However, it is unclear how aging impacts the production of monocyte subsets in the bone marrow and to what extent sexual dimorphism affects that. The current study aimed to fill this gap in the literature using male and female mouse models of aging.

We observed elevated numbers of peripheral monocytes and bone marrow myeloid progenitors in both male and female old (24–30 month) mice, indicating increased myelopoiesis during aging in both sexes. scRNAseq analysis revealed increased expression of DCMo signature genes such as *H2* genes encoding MHCII molecules and *Cd74* by bone marrow classical monocytes from old mice of both sexes, as well as elevated expression of the inflammatory regulator *Aw112010*. Consistent with this, flow cytometry analysis showed a higher proportion and elevated number of classical monocytes expressing MHCII and CD74 proteins (DCMo) in the bone marrow of old mice. Moreover, old bone marrow MDPs and bone marrow classical monocytes yielded proportionally more CD11c^+^ MHCII^+^ moDC in GM‐CSF cultures. Finally, using mouse macrophages, we found that *Aw112010* regulates the induction of MHCII expression, which provides a potential mechanism for MHCII upregulation during monocyte aging. Together, these data indicate that during aging there is increased production of DCMo and moDC by MDPs in both male and female mice. Consistent with this, we also observed increased MHCII expression by classical monocytes in humans during healthy aging.

## 2 RESULTS

### 2.1 Peripheral myeloid cells are increased in old mice of both sexes

**FIGURE 1:**
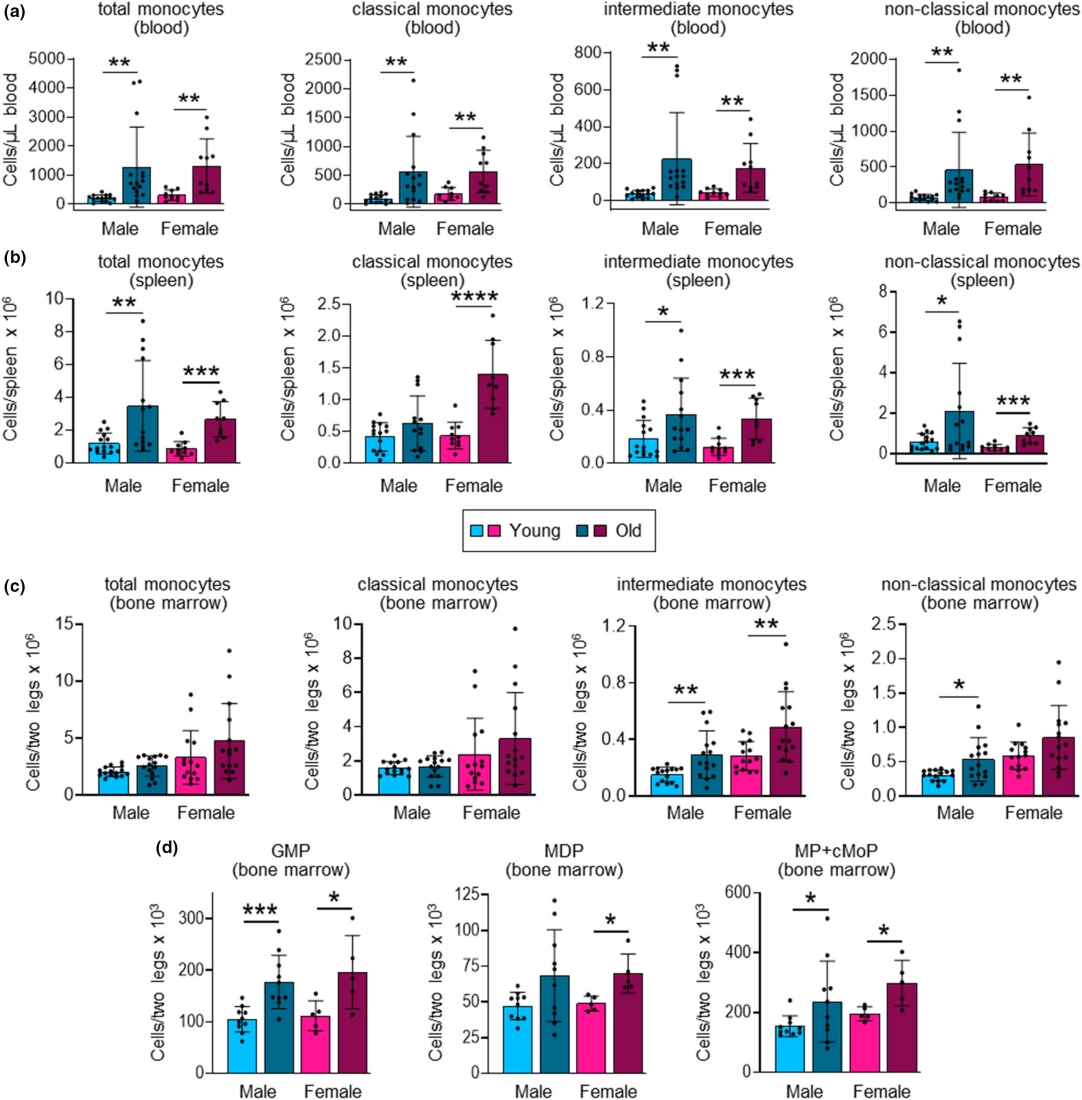
Aging increases peripheral monocytes and monocyte progenitors in the bone marrow. (a, b) The number of total monocytes (c‐Kit^−^ CD11b^+^ Ly6G^−^ CD115^+^ cells) and classical (Ly6C^hi^), intermediate (Ly6C^int^) and non‐classical (Ly6C^lo^) monocyte subsets in the blood (a) and spleen (b) of young (2–6‐month) and old (24–30 month), male and female mice was assessed by flow cytometry (see Figures S1A and S2A for gating strategies). (c, d) The number of total monocytes, and classical, intermediate and non‐classical monocyte subsets (c) and GMPs (Lin^−^ c‐Kit^+^ FcγR^hi^ Ly6C^−^ Flt3^−^ CD115^lo^ cells), MDPs (Lin^−^ c‐Kit^+^ FcγR^lo^ Ly6C^−^ Flt3^+^ CD115^hi^ cells) and monocyte‐committed progenitors (MP + cMoP; Lin^−^ c‐Kit^+^ FcγR^hi^ Ly6C^+^ Flt3^−^ CD115^hi^ cells) (d) in the bone marrow of young and old, male and female mice was assessed by flow cytometry (see Figure S3A,C for gating strategy). Data are presented as mean plus standard deviation of 10–15 mice (a, b), 14–15 mice (c) and 5–10 mice (d) in each group, and statistical significance was assessed by two‐tailed Student's *t*‐test (*p < 0.05, **p < 0.01, ***p < 0.001, ****p < 0.0001).

We first evaluated how aging impacts myeloid and lymphoid cell numbers in male and female mice by comparing young (2–6 month) and old (24–30 month) mice. The total number of monocytes in the circulation increased with age in both male and female mice (Figure 1a and Figure S1A). Further analysis of monocyte subsets revealed that the numbers of Ly6C^hi^ classical, Ly6C^int^ intermediate and Ly6C^lo^ non‐classical monocytes in the circulation also increased in both sexes (Figure 1a and Figure S1A). The total number of monocytes in the spleen was similarly higher in old mice of both sexes (Figure 1b and Figure S2A). There were also significantly more classical monocytes in the spleens of old female mice, and more intermediate and non‐classical monocytes in the spleens of old mice of both sexes (Figure 1b and Figure S2A).

We also assessed neutrophils, which share common progenitors with monocytes. Neutrophils were significantly increased in the blood of old mice of both sexes (Figure S1). We also observed significantly more splenic neutrophils in old female mice, and a trend towards to an increase in old male mice (Figure S2).

### 2.2 Bone marrow myeloid progenitors are increased in old mice of both sexes

Total and classical monocyte numbers in the bone marrow were not significantly higher in old mice of either sex (Figure 1c and Figure S3A). However, intermediate monocyte numbers were significantly increased in the bone marrow of old mice of both sexes, and non‐classical monocyte numbers were significantly higher in old male mice, with a trend towards an increase in old female mice (Figure 1c and Figure S3A). There was no difference in the number of bone marrow neutrophils between the young and old mice of either sex (Figure S3A,B). Taken together, these data show an aging‐associated increase in the number of peripheral myeloid cells (monocytes and neutrophils) in both sexes, without any notable sex differences, but a less striking effect on myeloid cell numbers in the bone marrow, perhaps reflecting increased release into the circulation. Indeed, previous studies have demonstrated elevated CCR2 expression by classical monocytes and higher serum levels of its ligand MCP‐1/CCL2 in both mice and humans (Puchta et al., 2016; Seidler et al., 2010; Villeda et al., 2011).

Analysis of lymphoid cells showed no differences in the number of T cells in the blood or spleen of young and old mice of either sex (Figures S1 and S2), but there were significantly more T cells in the bone marrow of old mice of both sexes (Figure S3A,B), consistent with accumulation of memory and other aging‐associated T cell subsets (Elyahu et al., 2019). In addition, young and old female mice had more bone marrow T cells than their male counterparts (Figure S3A,B), as previously reported (Hensel et al., 2019). We also observed a modest increase in the number of mature B cells in the circulation of old males and in the spleen of old females (Figures S1 and S2), but no difference between young and old mice of either sex in the number of pre/pro B cells or mature B cells in the bone marrow (Figure S3A,B).

Taken together, our analysis of myeloid and lymphoid cells is in agreement with previous reports of increased myeloid cell output in old mice due to myeloid‐biased hematopoiesis during aging (Ho et al., 2019; Puchta et al., 2016; Seidler et al., 2010). Consistent with this, we observed significant increases or trends towards an increase in the numbers of several subsets of myeloid progenitors in the bone marrow of old mice of both sexes, including multilineage myeloid progenitors—GMPs and MDPs—as well as monocyte‐committed progenitors (GMP‐derived MPs and MDP‐derived cMoPs) (Figure 1d and Figure S3C). Notably, both GMP and MDP numbers were elevated. We have previously demonstrated that monocytes arise independently from GMPs and MDPs (Yáñez et al., 2017), so this observation indicates that monocyte production via both pathways is likely increased during aging.

### 2.3 Aging increases the expression of genes associated with antigen presentation by the DCMo subset of classical monocytes in the bone marrow

**FIGURE 2:**
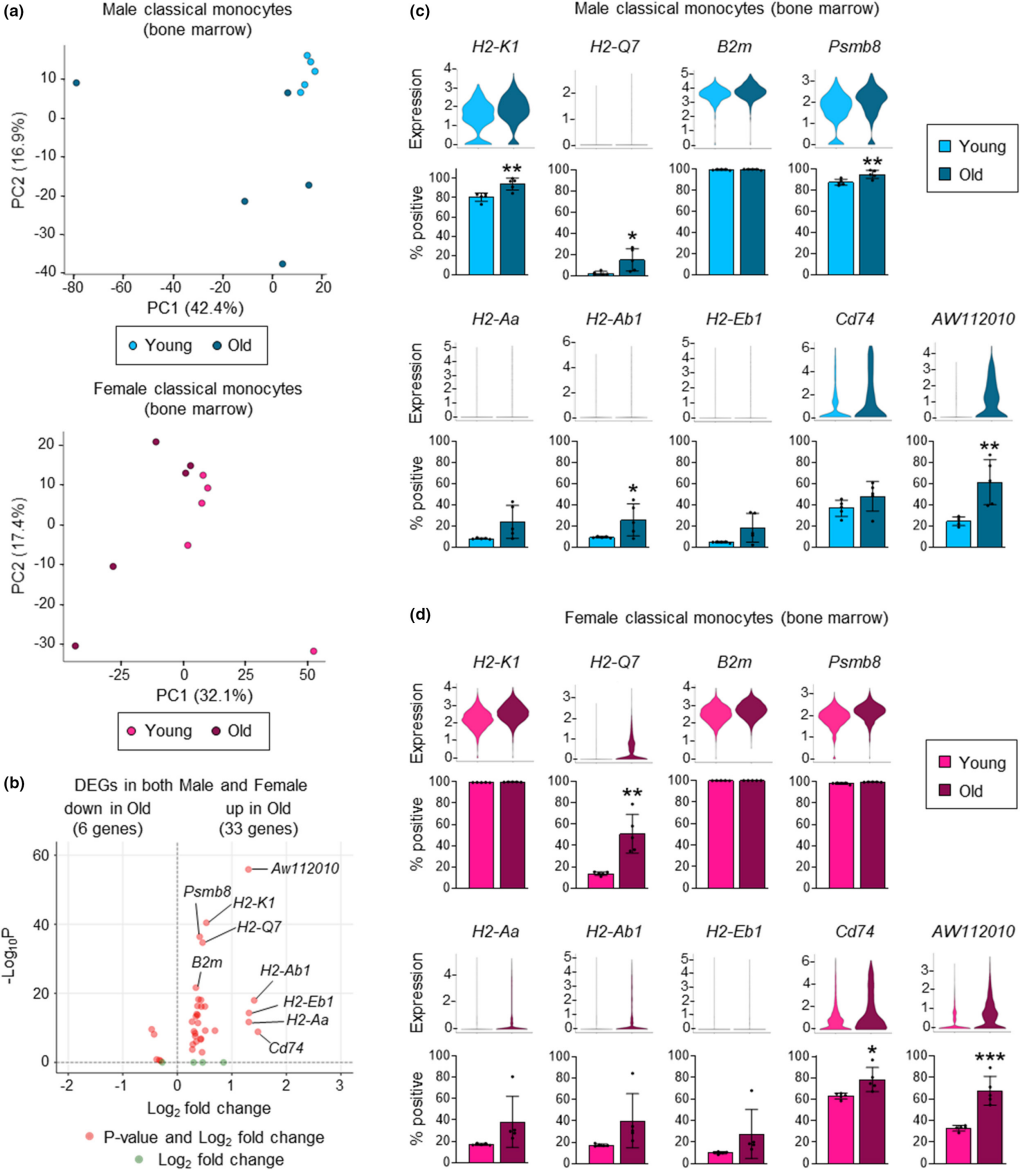
Aging increases the expression of genes associated with antigen presentation in bone marrow classical monocytes. scRNAseq analysis of classical (Ly6C^hi^) monocytes from the bone marrow of young and old, male and female mice (5 mice per group). (a) Principal component analysis of young and old, male (upper panel) and female (lower panel) mice. (b) Volcano plot of aging‐associated differentially expressed genes (DEGs; old vs. young) that are increased or decreased in both male and female mice. Aging‐associated DEGs were first defined separately using the male and female datasets (see Figure S4C–E) and then mean fold changes were calculated and Fisher's method was used to obtain combined adjusted *p* values. (c, d) Expression of DEGs (upper panels; all ‐Log_10_ P > 8) and percentage positive cells (lower panels) in young and old classical monocytes from male (c) and female (d) mice. Percentage positive cells are presented as mean plus standard deviation of 5 mice in each group, and statistical significance was assessed by two‐tailed Student's *t*‐test (*p < 0.05, **p < 0.01, ***p < 0.001, ****p < 0.0001).

To evaluate the impact of aging on monocyte function, we next performed scRNAseq of FACS‐sorted bone marrow classical (Ly6C^hi^) monocytes from young and old mice (male and female mice assessed separately; 5 mice per group with cell hashing to enable sample multiplexing and facilitate multiplet exclusion; Figure S4A,B). Principal component (PC) analysis showed clear separation of young and old monocytes in both the male and female datasets (Figure 2a).

We next analyzed differentially expressed genes (DEGs), focusing on genes that were upregulated or downregulated (*p* ≤ 0.05) in both males and females (Figure 2b and Figure S4C–E and Table S1A–D). Strikingly, these genes include: MHCI (*H2‐Q7*, *H2‐K1*) and MHCII genes (*H2‐Aa*, *H2‐Ab1*, *H2‐Eb1*) genes; *B2m*, which encodes the β2‐microglobulin subunit that combines with the MHCI chain to form the MHCI heterodimer; *Psmb8*, which encodes the β5i subunit of the immunoproteasome that digests peptides for loading on to MHCI molecules; and *Cd74*, which codes for the invariant chain that chaperones MHCII molecules to endosomes. Increased expression of most of the DEGs was due to both an increased proportion of cells expressing these genes and higher expression by individual cells (Figure 2c,d). Thus, healthy aging alters the steady‐state expression of several genes associated with MHCI‐ and MHCII‐mediated antigen presentation in bone marrow classical monocytes. Expression of *Aw112010* was also notably increased in both male and female monocytes (Figure 2b–d and Figure S4C–E and Table S1A–D).

**FIGURE 3:**
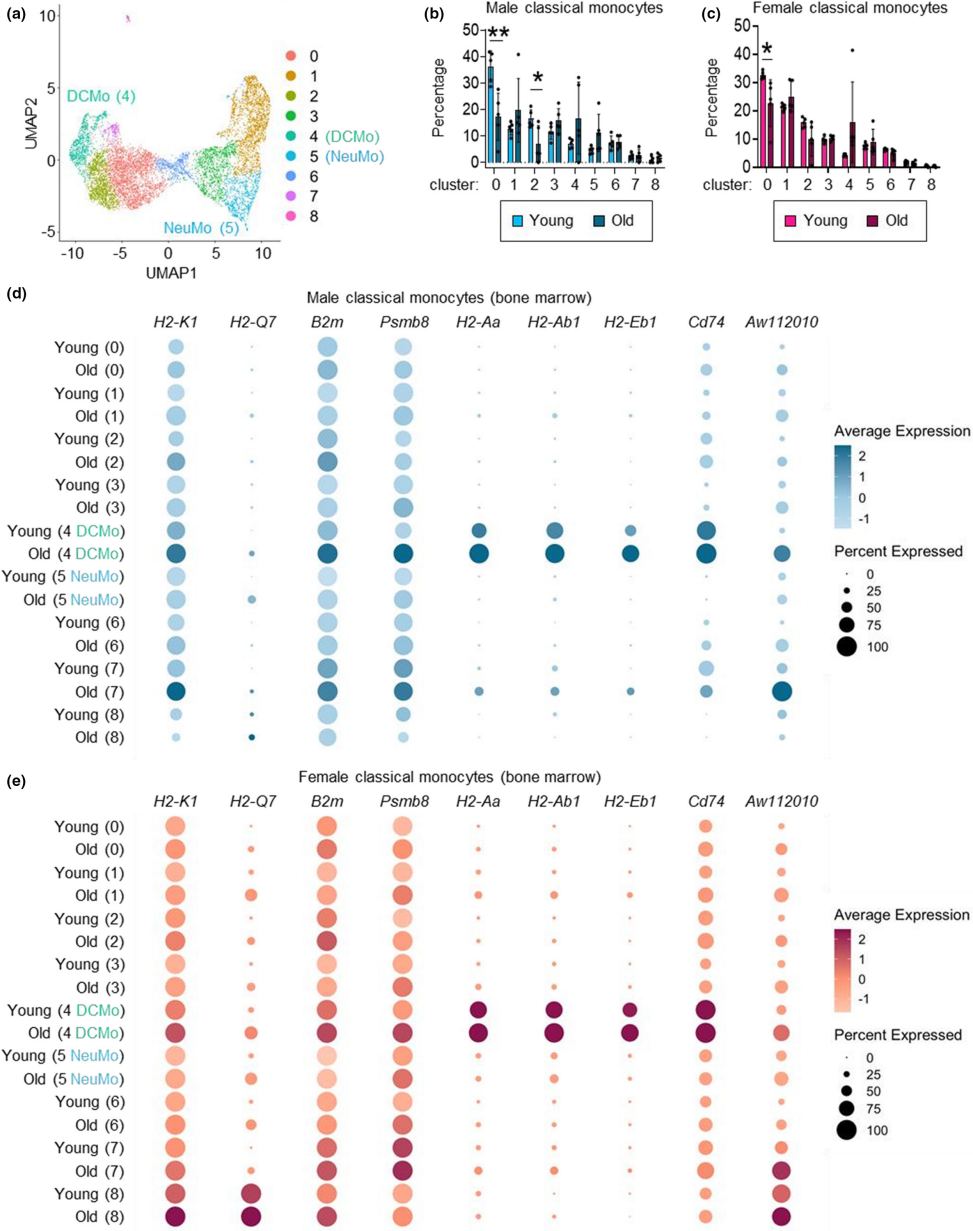
Aging increases DCMo gene expression. (a) UMAP visualization of classical monocytes from young and old, male and female mice assessed by scRNAseq (8279 cells, all groups). NeuMo are cluster 5, DCMo are cluster 4; see also Figure S5C,D). (b, c) Proportions of monocytes in each cluster in young and old, male (b) and female (c) mice. (d, e) Dot plots show DEG expression across monocyte clusters in young and old, male (d) and female (e) mice. Average gene expression by all cells in the cluster is indicated by the color intensity scale, and the size of the dot shows the percentage of cells expressing the gene.

We next evaluated subsets of classical monocytes by cell clustering. We identified nine clusters, which were present in all groups (young and old, male and female; Figure 3a and Figure S5A,B and Table S2). We have previously demonstrated that a subset of neutrophil‐like Ly6C^hi^ monocytes is produced by GMPs, and that MDPs give rise to moDC‐producing Ly6C^hi^ monocytes (Yáñez et al., 2017). We identified these subsets, which have been termed NeuMo and DCMo, respectively (Trzebanski & Jung, 2020), in our current dataset (Figure 3a and Figure S5C,D) using marker genes we previously defined (Yáñez et al., 2017). Compositional analysis of the clusters showed no change in NeuMo (cluster 5) proportions during aging, but there was a trend towards an increase in DCMo (cluster 4) proportions in both males and females (Figure 3b,c). Moreover, one of the other monocyte clusters (cluster 0) was proportionally decreased in old mice compared with young mice in both sexes and another (cluster 2) decreased in old males (Figure 3b,c).

Next, we assessed the expression of the aging‐associated DEGs by the monocyte clusters (Figure 3d,e and Figure S6). Notably, the MHCII and associated genes—*H2‐Aa*, *H2‐Ab1*, *H2‐Eb1*, and *Cd74—*are signature genes of the MDP‐derived DCMo cluster. Expression of these genes was predominantly restricted to DCMo in young mice and further enhanced in old mice, reflecting increases in both the proportion of cells expressing the genes and their expression levels. The MHCI and associated genes—*H2‐K1*, *H2‐Q7*, *B2m*, and *Psmb8—*were more broadly expressed in young mice of both sexes, and their expression increased in all clusters during aging, but most notably in DCMo and cluster 7. *Aw112010* expression was relatively low in young mice but increased in all clusters in old mice, especially in DCMo and cluster 7. Taken together, these data show increased expression of DCMo signature genes and a few other more broadly expressed genes related to antigen presentation in classical monocytes from the bone marrow of both male and female old mice. In contrast, NeuMo signature genes were not differentially expressed between young and old monocyte clusters in either sex (Figure S7).

### 2.4 The proportion of classical monocytes expressing MHCII and CD74 proteins is increased in old mice

**FIGURE 4:**
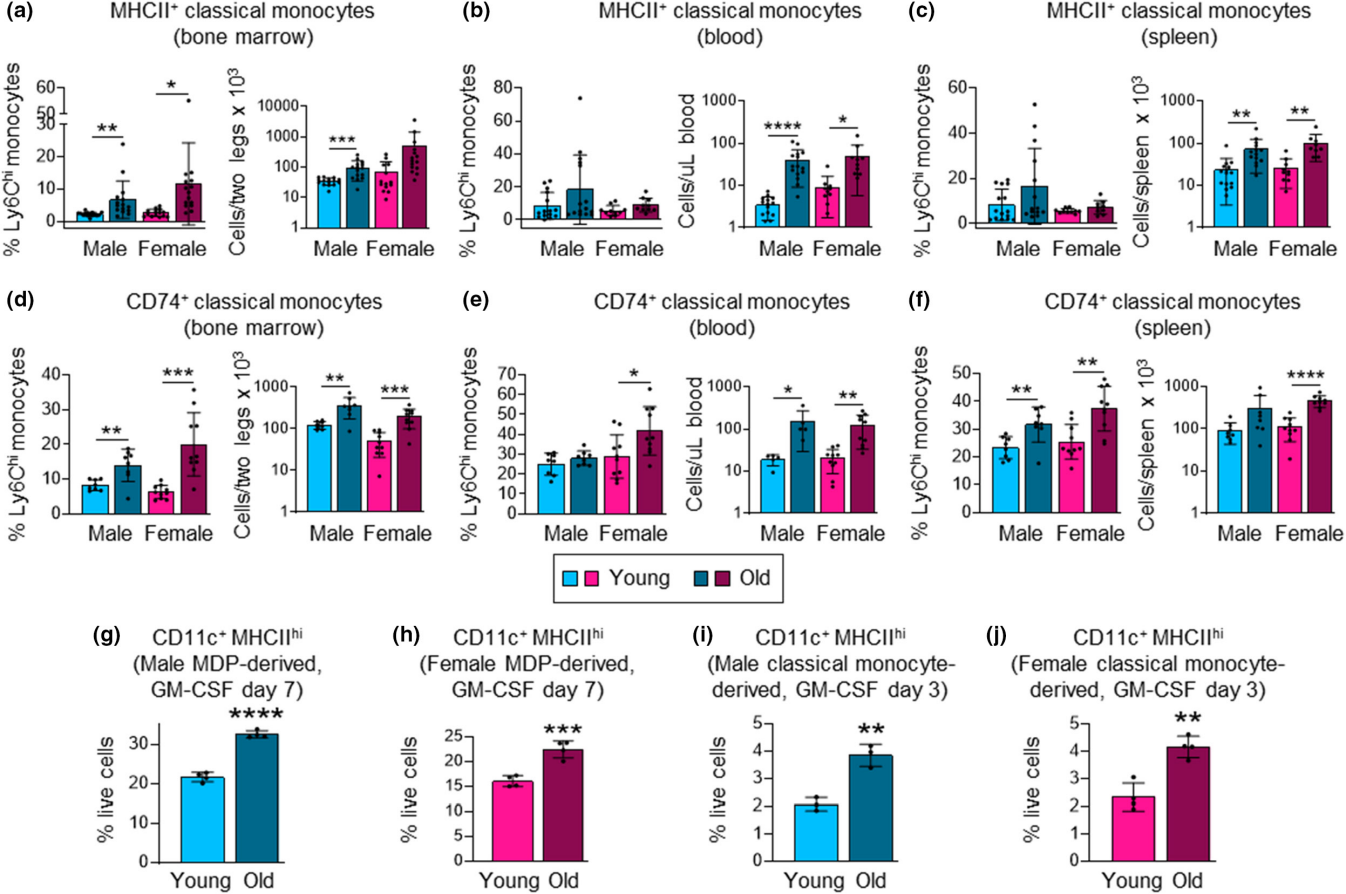
The proportion of classical monocytes expressing MHCII and CD74 proteins increases during aging, and old MDPs and classical monocytes yield more moDC. (a–f) The expression of surface MHCII and intracellular CD74 protein by classical monocytes from the bone marrow (a, d), blood (b, e) and spleen (c, f) of young and old, male and female mice was assessed by flow cytometry (see Figure S9A–C for gating). (g–j) MDPs and classical monocytes were FACS‐sorted from young and old, male and female mouse bone marrow and cultured with 20 ng/ml GM‐CSF. moDC (CD11c^+^ MHCII^hi^ cell) production was assessed by flow cytometry after 7 days for MDP cultures (g, h) and after 3 days for monocyte cultures (i, j). Data are presented as mean plus standard deviation of 10–15 mice (a–f), and 3–4 replicates of cultures derived from cells pooled from 3–5 mice in each group (h–j). Statistical significance was assessed by two‐tailed Student's *t*‐test (*p < 0.05, **p < 0.01, ***p < 0.001, ****p < 0.0001).

Since our scRNAseq analysis most strikingly revealed increased expression of genes encoding MHCII and CD74 by old classical monocytes, we next examined whether these differences are also reflected at the level of MHCII and CD74 proteins. We assessed surface MHCII and intracellular CD74 expression by classical monocytes from the bone marrow, blood, and spleen of young and old mice of both sexes by flow cytometry. The proportions and numbers of MHCII^+^ classical monocytes were very low in the bone marrow of young mice but increased in old mice of both sexes (Figure 4a and Figure S8A). The proportions and numbers of CD74^+^ classical monocytes similarly increased in the bone marrow of old mice, and there were also more MHCII^+^ and CD74^+^ classical monocytes in the blood and spleens of old mice of both sexes (Figure 4b–f and Figure S8A–C).

Taken together, the scRNAseq and protein expression data demonstrate that the increased proportion of classical monocytes expressing MHCII and CD74 in the circulation and spleen of old mice reflects elevated production of MDP‐derived DCMo in the bone marrow, rather than upregulation of MHCII and CD74 after differentiation.

We also evaluated MHCII and CD74 expression by intermediate and non‐classical monocytes and observed statistically significant increases or trends toward significant increases in the numbers of MHCII^+^ and CD74^+^ intermediate and non‐classical monocytes in the bone marrow, blood, and spleen of both males and females (Figure S8D–O).

### 2.5 Aging promotes moDC production by MDPs and classical monocytes

Next, we examined whether the biased DCMo production results in increased moDC potential by evaluating moDC production by MDPs. Upon in vitro culture of MDPs from young and old, male and female mice with GM‐CSF for 7 days, significantly higher proportions of moDC were observed among the progeny of old MDPs compared with the progeny of young MDPs (Figure 4g,h and Figure S9A,B). Classical monocytes from old mice also produced proportionally more moDC than their young counterparts in GM‐CSF cultures (Figure 4i,j and Figure S9C,D). These data further validate our observation of increased MDP‐derived DCMo in the bone marrow of old mice and indicate that aging promotes moDC differentiation from MDPs via increased production of DCMo.

### 2.6 *Aw112010* lncRNA regulates MHCII in mouse macrophages

Our scRNAseq analysis also revealed increased expression of *Aw112010* by old classical monocytes. *Aw112010* was initially defined as a long non‐coding RNA (lncRNA), but subsequently shown to possess a non‐canonical open reading frame that is translated (Jackson et al., 2018). Indeed, it can act as both a lncRNA and a protein to regulate cytokine production (Jackson et al., 2018; Yang et al., 2020). We therefore examined whether *Aw112010* also regulates MHCII and CD74 expression.

**FIGURE 5:**
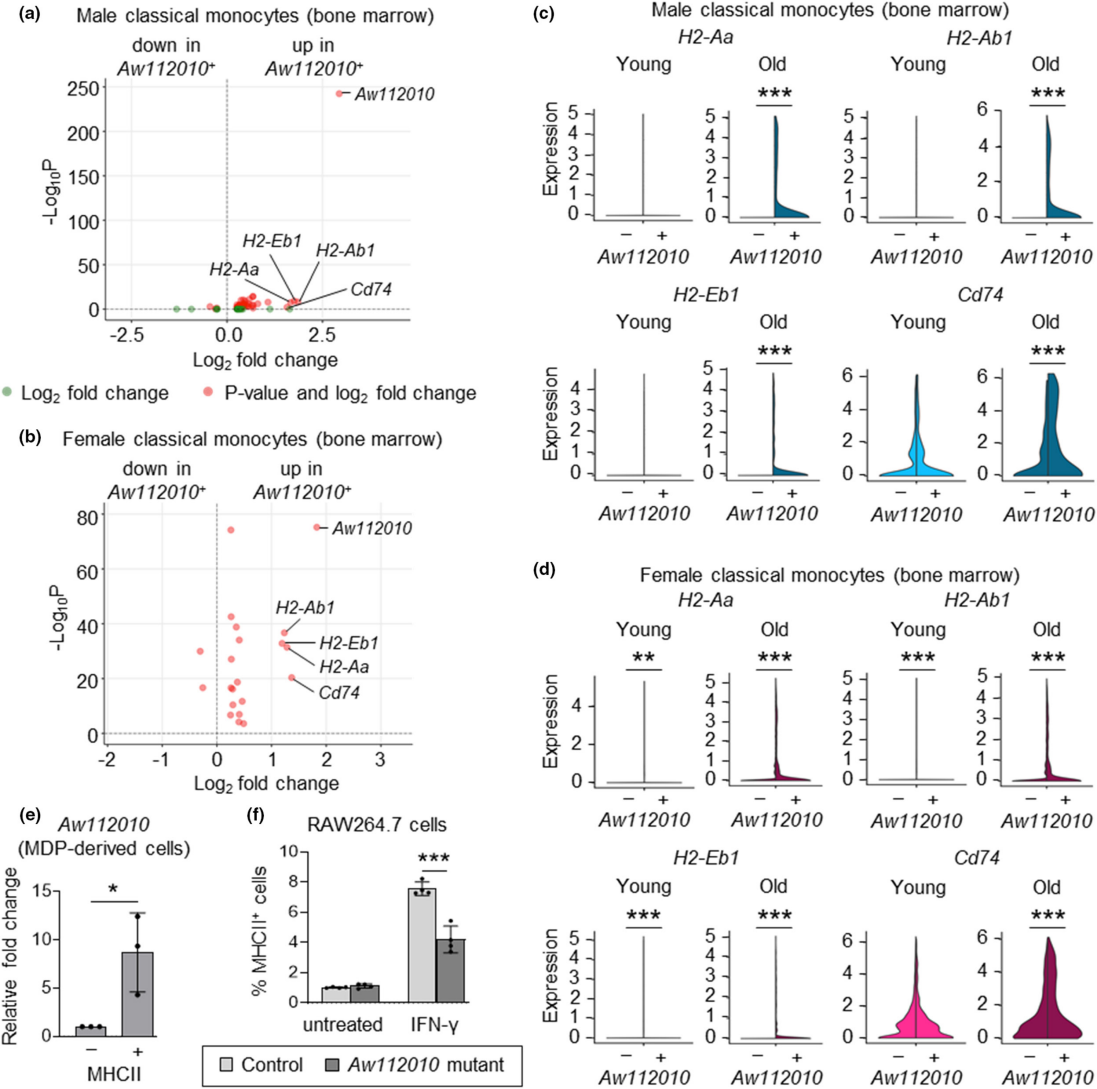
*Aw112010* regulates MHCII expression by mouse macrophages. (a, b) Volcano plots show DEGs between classical monocytes (young and old combined) expressing and lacking *Aw112010* (*Aw112010* ^+^ and *Aw112010* ^−^, respectively) in male (a) and female (b) mice. Genes with Log_2_ fold change ≥1 are labeled. (c, d) Violin plots show the expression of *H2* genes and *Cd74* by classical monocytes expressing and lacking *Aw112010* (*Aw112010* ^+^ and *Aw112010* ^−^, respectively) in young and old, male (c) and female (d) mice. (e) qRT‐PCR measurement of *Aw112010* expression in FACS‐sorted MHCII^+^ and MHCII^−^ cells from 7‐day GM‐CSF cultures of young mouse bone marrow MDPs (see Figure S9E for gating). Data are presented as mean plus standard deviation of three replicates of MDP cultures derived from cells pooled from five mice. (f) Control and *Aw112010* mutant RAW264.7 cells were treated with IFN‐γ (10 ng/ml) for 24 h and MHCII expression was assessed by flow cytometry (see Figure S9F for gating strategy). Data are presented as mean plus standard deviation of four replicates of RAW264.7 cell cultures, which are representative of three independent experiments. Statistical significance (c–f) was assessed by two‐tailed Student's *t*‐test (*p < 0.05, **p < 0.01, ***p < 0.001).

We first compared classical monocytes expressing or lacking *Aw112010* in our scRNAseq dataset. DEG analysis showed higher expression of *H2‐Aa*, *H2‐Ab1*, *H2‐Eb1*, and *Cd74* in cells expressing *Aw112010* (at least 1 raw count per cell) in both sexes (Figure 5a–d and Table S3). Furthermore, qRT‐PCR of MDP‐derived cells in day 7 GM‐CSF cultures showed higher expression of *Aw112010* by MHCII^+^ cells compared with MHCII^−^ cells (Figure 5e and Figure S9E).

We next directly assessed whether the *Aw112010* lncRNA regulates surface MHCII expression using RAW264.7 mouse macrophages from which a fragment of the non‐coding sequence downstream of the coding sequence had been deleted (Yang et al., 2020). RAW264.7 macrophages express low levels of MHCII, so we stimulated them with IFN‐γ to promote its expression and observed lower MHCII induction in *Aw112010* mutant cells than control cells (Figure 5f and Figure S9F). Together, these data identify *Aw112010* lncRNA as a regulator of MHCII expression and suggest that increased *Aw112010* lncRNA levels may underlie elevated MHCII gene expression in old monocytes.

### 2.7 Human classical monocytes also express more MHCII during healthy aging

**FIGURE 6:**
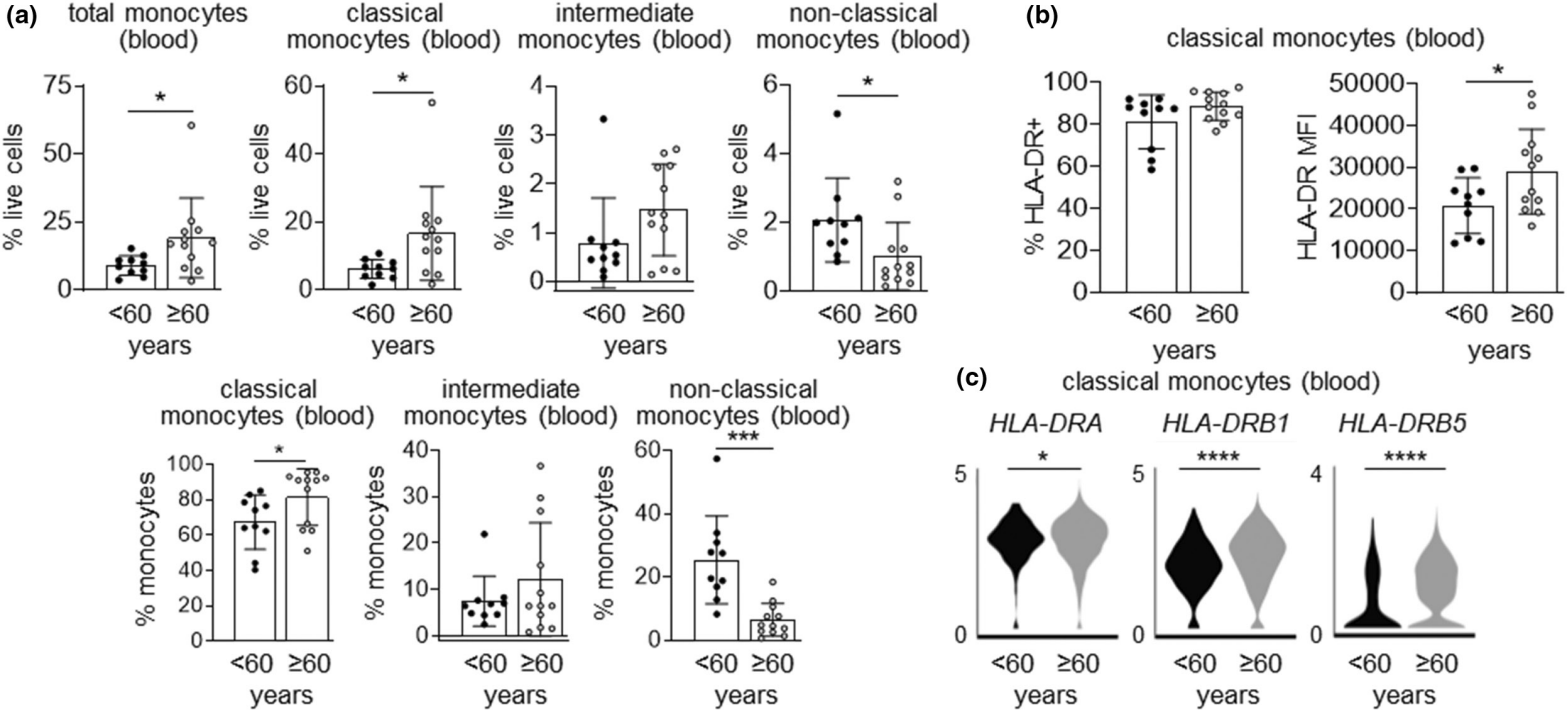
Human classical monocytes also express more MHCII during aging. (a) The proportion of total monocytes (CD3^−^ CD20^−^ CD56^−^ cells that are positive for CD14 and/or CD16), and classical (CD14^+^ CD16^−^ cells), intermediate (CD14^+^ CD16^+^ cells) and non‐classical (CD14^−^ CD16^+^ cells) monocyte subsets in the peripheral blood of younger (<60 years; median age 46) and older (≥60 years; median age 70) human volunteers was measured by flow cytometry (see Figure S10A for gating strategy and Figure S10F for separate analysis of males and females). (b) Flow cytometry measurement of HLA‐DR expression by peripheral blood classical monocytes from younger and older individuals (see Figure S10G for separate analysis of males and females). Flow cytometry data are presented as mean plus standard deviation of 10 younger (three male and seven female) and 12 older (five male and seven female) subjects. (c) Violin plots show the expression of *HLA‐DR* genes by peripheral blood classical monocytes from younger and older individuals (4 per group, all male) assessed by scRNAseq (see Figure S10E for classical monocyte identification). Statistical significance was assessed by two‐tailed Student's *t*‐test (*p < 0.05, ***p < 0.001, ****p < 0.0001).

Finally, we assessed the composition of monocyte subsets and expression of HLA‐DR (human MHCII) by classical monocytes from the blood of healthy younger (<60 years) and older (≥60 years) adult humans (Lewis et al., 2021). Consistent with our mouse studies, the proportions of total monocytes and classical monocytes in the circulation were significantly higher in older individuals (male and female combined; Figure 6a and Figure S10A,B). There was no difference in the proportion of intermediate monocytes between younger and older subjects, whereas non‐classical monocytes were significantly lower in older individuals (Figure 6a and Figure S10B). The proportion of HLA‐DR^+^ classical monocytes was not significantly affected by aging (most were already positive in younger individuals), but the level of HLA‐DR protein was higher in older individuals (Figure 6b and Figure S10C). Consistent with this and previous reports by others (Huang et al., 2021; Metcalf et al., 2017), scRNAseq analysis revealed increased expression of HLA‐DR genes, most notably *HLA‐DRB5*, by classical monocytes from older individuals (Figure 6c and Figure S10D,E). Analysis of males and females separately showed a similar trend in monocyte composition and HLA‐DR expression by classical monocytes during aging in both sexes, although most differences did not reach statistical significance, likely due to low sample numbers (Figure S10F,G).

## 3 DISCUSSION

Collectively, our data indicate that while aging increases the production of classical monocytes by both GMPs and MDPs in mice, there is a specific increase in the production of MDP‐derived MHCII^+^ classical monocytes (DCMo), which results in increased potential for moDC production. We also observed increased MHCII expression by classical monocytes in humans during healthy aging, although most human peripheral blood monocytes constitutively express MHCII molecules and their origins (GMP and/or MDP‐derived) have not been defined.

Mechanistically, our data demonstrate that increased expression of the lncRNA *Aw112010*, which promotes MHCII expression by macrophages, may underlie increased moDC production during aging in mice. *Aw112010* regulates cytokine production via both coding and non‐coding functions (Jackson et al., 2018; Yang et al., 2020). Here, we show that the non‐coding function of *Aw112010* is specifically important for MHCII induction in mouse macrophages. Increased *Aw112010* lncRNA expression may therefore also underlie elevated *H2* gene expression in classical monocytes during aging. The *Aw112010* lncRNA has previously been shown to inhibit IL‐10 and promote IL‐6 production by LPS‐stimulated RAW264.7 macrophages (Yang et al., 2020). Moreover, *Aw112010* suppresses IL‐10 production by T cells by interacting with the histone demethylase KDM5A, which results in decreased H3K4 methylation at the IL‐10 gene locus (Yang et al., 2020). *Aw112010* may similarly regulate H2 gene induction in aging monocytes via direct epigenetic effects, or alternatively it may promote autocrine signaling via elevation of basal production of inflammatory cytokines that indirectly promote H2 gene expression (Ho et al., 2019) or via reduction of IL‐10. It may also act via the protein encoded by its non‐canonical open reading frame. Indeed, the Aw112010 protein mediates LPS‐induced IL‐12 production by macrophages (Jackson et al., 2018).

An *Aw112010* homolog has not been found in humans, but HLA gene expression may be controlled by a related regulatory program. It will therefore be important to define mechanisms underlying DCMo production more thoroughly, including the *Aw112010* regulatory network. Aging has been shown to induce epigenetic modifications in human peripheral blood monocytes (Cheung et al., 2018; Reynolds et al., 2014; Shchukina et al., 2021). Our demonstration of increased moDC production in old MDP cultures indicates that old MDPs are intrinsically programmed to produce more DCMo in old mice. One potential mechanism that might explain changes in monocyte subset composition and gene expression profiles is clonal hematopoiesis, although the impact of clonal hematopoiesis in mice is somewhat controversial. A recent study reported that old mice can acquire clonal hematopoiesis mutations, but there is limited expansion of mutant clones during the lifetime of a mouse (Chin et al., 2022), so it seems unlikely that this mechanism is responsible for increased DCMo and moDC production during aging. It will, however, be interesting to determine whether epigenetic and metabolic changes in monocytes and their progenitors underlie increased DCMo and moDC production.

Despite demonstrating increased expression of MHC molecules and other proteins associated with antigen presentation by old classical monocytes, our data do not necessarily indicate that old monocytes have an increased capacity for antigen presentation. Studies to determine how aging impacts antigen uptake and processing, upregulation of co‐stimulatory molecules and production of T cell‐polarizing cytokines by classical monocytes, as well as their capacity to stimulate T cell proliferation and differentiation, are required to determine whether the DCMo subset and the moDC they produce are functionally competent for T cell activation. It is possible that the increased expression of antigen presentation‐associated genes simply reflects their inflammatory state, which may result in functional impairment. For example, the bone marrow of old mice contains an increased proportion of MHCII^+^ inflammatory macrophages, which show impaired phagocytosis of senescent neutrophils and promote myeloid bias in hematopoietic stem cells via IL‐1β (Frisch et al., 2019). The relationship between these macrophages and the DCMo, specifically whether the macrophages are DCMo‐derived, also remains to be defined. Moreover, it will be interesting to determine whether atherosclerotic plaque‐associated macrophages, which have increased expression of MHCII and CD74 genes, are derived from DCMo, and whether there is any link between increased DCMo and the risk of atherosclerosis during aging (Lin et al., 2019; Tyrrell & Goldstein, 2021).

We previously showed that non‐classical monocytes also arise from both GMPs and MDPs (Yáñez et al., 2017). Since non‐classical monocytes are derived from classical monocytes, presumably there may be at least as many non‐classical monocyte subsets as there are classical monocyte subsets. In the current study, we observed increased numbers of MHCII^+^ non‐classical monocytes in the bone marrow of old mice. In future studies, it will be important to define the origins of non‐classical monocyte subsets, including whether MHCII^+^ non‐classical monocytes are derived from DCMo. scRNAseq profiling of non‐classical monocytes could also be used to reveal changes in subset composition and gene expression during aging.

Sexual dimorphism impacts the innate immune system, rendering males more susceptible to severe infections and females more prone to autoimmune disorders (Jaillon et al., 2019; Klein & Flanagan, 2016; Sampathkumar et al., 2020). Peripheral blood monocytes have been shown to exhibit higher expression of genes associated with immune cell activation in women compared with men in the context of chronic low‐grade inflammation (So et al., 2021). We did not directly compare male and female monocytes in the current study because we assessed male and female monocytes in separate experiments, but we did observe similar changes in monocyte subsets in the bone marrow, blood, and spleen during healthy aging in both males and females, and we found some shared gene expression changes, including increased MHCI and MHCII gene expression. Sexual dimorphism during monocyte aging will be an important topic for future studies.

In conclusion, the current study reveals that healthy aging promotes monocyte production via the GMP‐ and MDP‐derived pathways in both sexes, but that production of the DCMo subset of classical monocytes by MDPs is specifically increased. Inflammation induced by commensal microbe‐derived products that leak from the gut into the circulation due to a cycle of tissue macrophage inflammation, microbial dysbiosis and increased intestinal permeability during aging (Bosco & Noti, 2021; Ragonnaud & Biragyn, 2021; Thevaranjan et al., 2018) may underlie the increased production and altered functions of monocytes during aging. Future studies to define the mechanisms underlying such alterations in monocyte production and function will improve our understanding of the role of monocytes and their progenitors in aging‐associated disorders such as myelodysplasia and cardiovascular diseases.

## 4 MATERIALS AND METHODS

### 4.1 Experimental design

In this study, we used a combination of approaches (flow cytometry, single‐cell RNA sequencing, and differentiation assays) to profile monocytes, their progenitors and other immune cells from the bone marrow, blood and spleen of young and old mice, as well monocytes from the blood of healthy younger and older humans.

### 4.2 Mice

Wild‐type C57BL/6 mice were purchased from The Jackson Laboratories and maintained at Cedars‐Sinai Medical Center animal facility. Young (2–6 months old) and old (24–30 months old) male and female mice were used. IACUC regulations were followed to perform all procedures.

### 4.3 Flow cytometry and MACS and FACS sorting

Antibodies used for flow cytometry and FACS sorting are listed in Table S4A. To assess myeloid and lymphoid cells and monocyte expression of MHCII, cells were stained with antibodies against c‐Kit‐BV650, Ly6G‐BV421, CD11b‐BUV395, CD115‐PE, Ly6C‐PerCP/Cy5.5, CD3εAPC, B220‐FITC, and MHCII‐AF700. To assess intracellular CD74 in monocytes, cells were stained with antibodies against c‐Kit‐BV650, Ly6G‐BV421, CD11b‐BUV395, CD115‐PE, and Ly6C‐PerCP/Cy5.5 followed by fixation, permeabilization, and staining with antibody against CD74‐AF647. For identification of myeloid progenitors, lineage‐negative (Lin^−^) cells were enriched by autoMACS using a direct lineage cell depletion kit (containing antibodies against CD5, CD45R (B220), CD11b, Gr‐1 (Ly‐6G/C), 7–4 and Ter‐119), and stained with antibodies against c‐Kit‐BV421, FcγR‐BV510, Flt3‐PE, CD115‐APC, and Ly6C‐APC/Cy7 following prestaining with Zombie Red viability dye.

To FACS sort bone marrow classical monocytes for scRNAseq, antibodies against Ly6G‐BV421 (to exclude neutrophils), CD11b‐APC, CD115‐PE, and Ly6C‐PerCP/Cy5.5 were used. To FACS sort classical monocytes for culture, bone marrow cells were enriched for monocytes by depleting neutrophils, T cells and B cells using biotin conjugated antibodies against Ly6G, CD3ε, and B220 and anti‐biotin magnetic beads prior to staining with antibodies against c‐Kit‐BV421 (to exclude progenitors), Ly6G‐APC (to exclude neutrophils), CD11b‐PE, CD115‐AF488 and Ly6C‐PerCP/Cy5.5. To FACS sort MDPs, bone marrow Lin^−^ cells were stained with antibodies against c‐Kit‐BV421, FcγR‐BV510, CD34‐AF647, Flt3‐PE, CD115‐AF488 and Ly6C‐APC/Cy7. For assessment of moDC in culture, cells were stained with Zombie Violet viability dye followed by antibodies against CD11c‐BV510 and MHCII‐AF700.

Where applicable, cells were incubated with Fc block prior to staining to prevent non‐specific antibody binding. For progenitor identification, cells were stained with antibodies against FcγR prior to staining with the other antibodies to prevent non‐specific binding. Flow cytometry was performed using an LSRFortessa (BD Biosciences) and data were analyzed with FlowJo 10.7.1. FACS sorting was performed using an Influx or FACS Aria III cell sorter (both from BD Biosciences).

### 4.4 Cell culture

FACS‐sorted monocytes and MDPs were cultured in RPMI 1640 medium supplemented with GM‐CSF (20 ng/ml), penicillin (50 U/ml), streptomycin (50 U/ml), L‐glutamine (2 mM), and FBS (10% v/v). Control and *Aw112010* mutant RAW264.7 cell lines (Yang et al., 2020) were maintained in RPMI 1640 and stimulated with IFN‐γ (10 ng/ml) in fresh medium.

### 4.5 Mouse monocyte isolation for Single‐Cell RNA sequencing (scRNAseq)

Bone marrow cells from young and old mice (5 mice in each group) were barcoded with TotalSeq hashtag antibodies (Table S4B), pooled, and classical Ly6C^hi^ monocytes (Ly6G^−^CD11b^+^CD115^+^Ly6C^hi^ cells) were isolated by FACS sorting. Male (young—14 weeks, old—27 months) and female (young—15 weeks, old—26.5 months) mice were processed separately.

### 4.6 scRNAseq library construction, sequencing and data analysis

Single‐cell RNAseq libraries were prepared using Single Cell 3′ v3.1 Reagent Kits User Guide (10× Genomics). Cells were loaded on a Chromium Controller instrument (10× Genomics) to generate single‐cell Gel bead‐in‐EMulsions (GEMs). GEM‐RT was performed in a Veriti 96‐well thermal cycler (Thermo Fisher Scientific). The cDNA was amplified and cleaned up with SPRIselect Reagent Kit (Beckman Coulter). Indexed sequencing libraries were constructed using Chromium Single‐Cell 3′ Library Kit and the barcoded sequencing libraries were quantified by qPCR using the Collibri Library Quantification Kit (Thermo Fisher Scientific).

Libraries were sequenced on a NovaSeq 6000 (Illumina) as per the Single Cell 3′ v3.1 Reagent Kits User Guide, with a sequencing depth of ~40,000 reads/cell.

The demultiplexed raw reads were aligned to the transcriptome using STAR (version 2.5.1) (Dobin et al., 2013) with default parameters and mouse mm10 transcriptome reference from Ensembl version 84 annotation, containing all protein coding and long non‐coding RNA genes. Expression counts for each gene in all samples were collapsed and normalized to unique molecular identifier (UMI) counts using Cell Ranger software version 4.0.0 (10× Genomics).

Data analysis was performed in R using Seurat v4. Datasets were processed using cell hashing to demultiplex samples and identify doublet and negative cells. Hashtag count data was normalized using CLR normalization. Samples were demultiplexed using the “HTODemux” function with default parameters, and negative and doublet cells were filtered out. For the female dataset, a small subset of cells was filtered out as they failed to cluster with their respective hashtags. Cells with greater than 200 expressed genes and <5% of mitochondrial genes were selected for downstream analysis. Datasets were normalized using SCTransform and mitochondrial gene expression was regressed out. The male and female datasets were integrated using the “IntegrateData” function after selecting 3000 integration features. Principal Component Analysis (PCA), dimensionality reduction using UMAP (using the first 20 principal components), and clustering were run on the integrated male and female dataset. For clustering, resolution was set to 0.3 to generate a total of nine clusters. Differential expression analysis for comparison of young and old monocytes was performed using the Wilcoxon‐test and the “FindMarkers” function. Aging‐associated differentially expressed genes (DEGs) were identified separately in the male and female datasets, and genes similarly upregulated or downregulated in both datasets were evaluated by calculating average fold changes in expression and using Fisher's method to obtain combined adjusted *p* values (with downsampling of the female dataset to evaluate similar numbers of male and female monocytes). Cluster markers were identified in the integrated dataset using the “FindAllMarkers” function with a minimum 25% and a log_2_fold change threshold of 0.25. Sample‐level PCA plots were generated by finding the average expression of the genes in each sample using the “AverageExpression” function. The dataset of averaged expression values was then processed using the standard Seurat workflow of data normalization, finding variable features, scaling data, and PCA using default values.

### 4.7 qRT‐PCR

A Power SYBR Green Cells‐to‐CT kit was used to isolate RNA and prepare first‐strand cDNA from FACS‐sorted MDP‐derived MHCII^+^ and MHCII^−^ cells. The Power SYBR Green PCR Master Mix and custom‐designed primers (Table S4C) were used to perform quantitative PCR.

Relative gene expression was measured using *18s rRNA* as a reference transcript.

### 4.8 Human monocyte datasets

The human monocyte datasets were obtained using blood samples collected from 23 healthy donors (three younger male, seven younger female, five older male and seven older female) (Lewis et al., 2021). The study was approved by the University of California Irvine Institutional Review Boards, and informed consent was obtained from all enrolled subjects. Samples were stratified by age: <60 years was categorized as younger, and ≥60 years was categorized as older. All samples were evaluated by flow cytometry. The median ages were 46 and 70 for the younger and older subjects, respectively. Four samples per group were evaluated by scRNAseq, with median ages of 35 and 78, respectively. PBMC isolation, flow cytometry, and scRNAseq analysis were performed as previously described (Lewis et al., 2021).

### 4.9 Statistical analysis

Statistical analyses of flow cytometry data were performed using two‐tailed Student's *t*‐tests in Prism 8.0 software (Graph‐Pad Inc), and differences with *p* ≤ 0.05 were considered significant. Statistical analyses of scRNAseq data are described above and in the Figure Legends.

## AUTHOR CONTRIBUTIONS

PKB and HSG designed the project; PKB and JES performed the mouse experiments and analyses and SK assisted them; DW, YW, and BAB assisted JES and HSG with the scRNAseq analysis; XY, PSN, and MN made the mutant *Aw112010* cell line; SAL and IM performed the human study and analyses; PKB and HSG wrote the manuscript; all authors edited and/or approved the manuscript.

## CONFLICT OF INTEREST

The authors declare that they have no competing interests.

## Supporting information


Figures S1–S10

Tables S1–S4

## Data Availability

The mouse and human scRNAseq datasets are available in GEO (GSE207063) and SRA (PRJNA727024) respectively.
