# Supplementary material for: Production of MHCII‐expressing classical monocytes increases during aging in mice and humans
Source: Aging Cell. 2022 Aug 30;21(10):e13701. doi: 10.1111/acel.13701 (PMC9577948; doi:10.1111/acel.13701)
Supplement: Supplementary file 1 — Figures S1–S10 [file ACEL-21-e13701-s001.pdf]

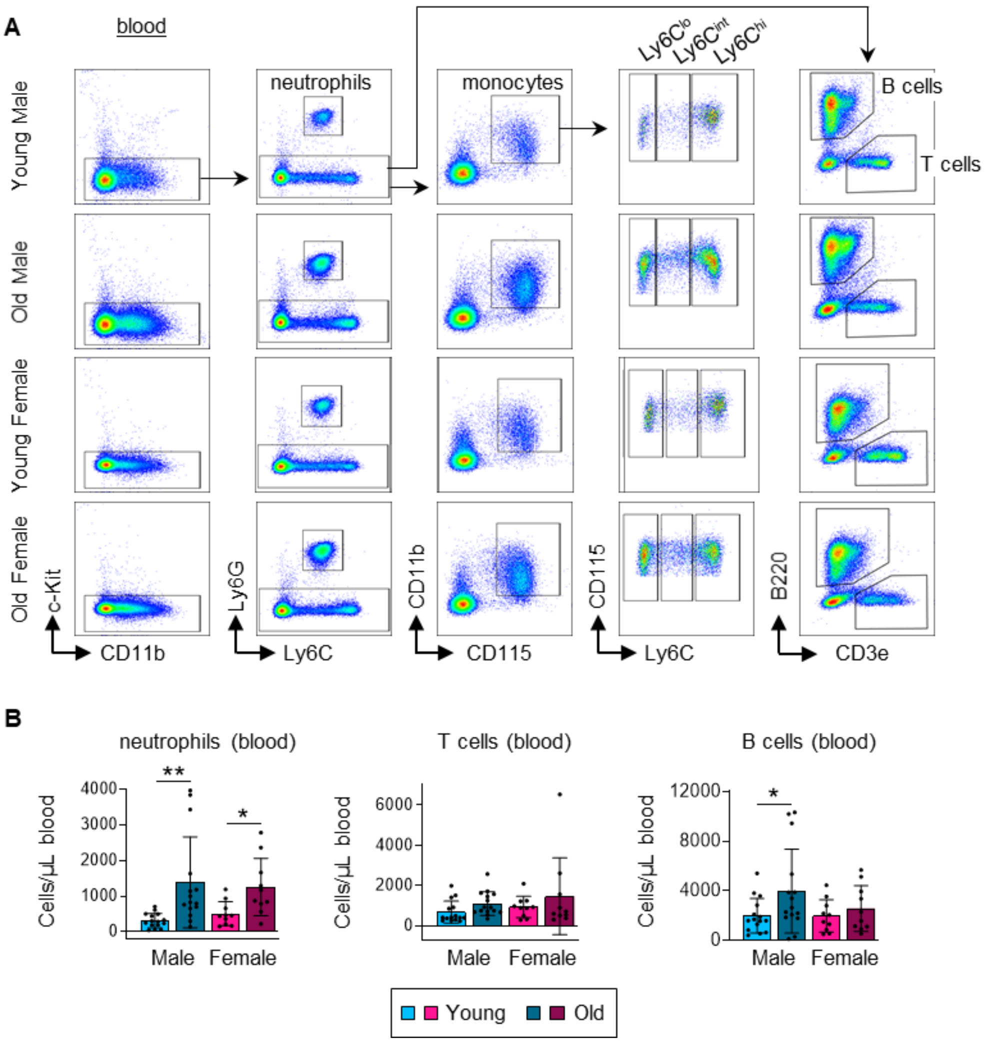

Fig. S1. Evaluation of myeloid and lymphoid cells in the blood. (A) Flow cytometry gating of peripheral blood cells for the identification of neutrophils (c-Kit^{-} Ly6G^{+} Ly6C^{int} cells), total monocytes (c-Kit^{-} CD11b^{+} Ly6G^{-} CD115^{+} cells), monocyte subsets (classical Ly6C^{hi}, intermediate Ly6C^{int}, and non-classical Ly6C^{lo}), T cells (c-Kit^{-} Ly6G^{-} B220^{-} CD3e^{+} cells) and B cells (c-Kit^{-} Ly6G^{-} CD3e^{-} B220^{+} cells). (B) The number of neutrophils, T cells and B cells in the blood of young and old, male and female mice was assessed by flow cytometry. Data are presented as mean plus standard deviation of 10-15 mice in each group, and statistical significance was assessed by two-tailed Student's t test (*$p < 0.05$, **$p < 0.01$).

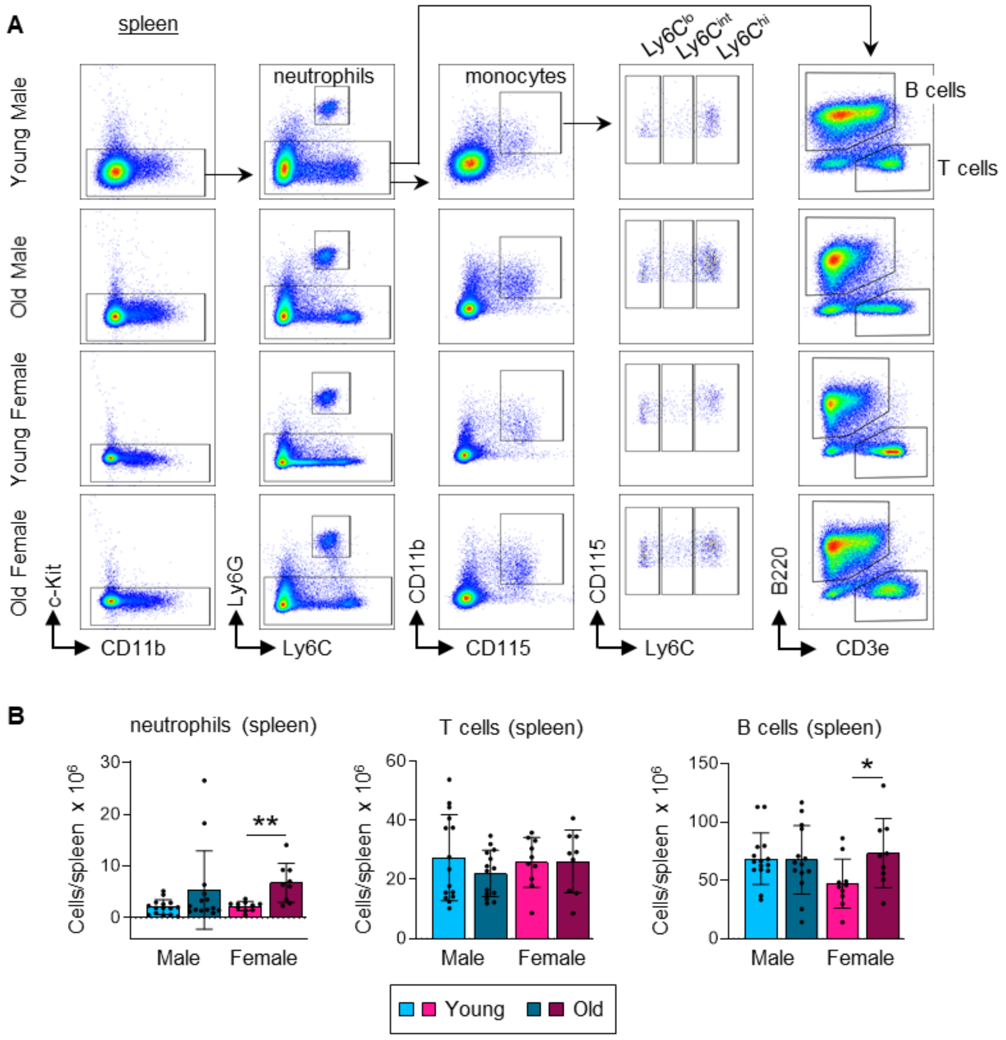

Fig. S2. Evaluation of myeloid and lymphoid cells in the spleen. (A) Flow cytometry gating of splenic cells for the identification of neutrophils, total monocytes, monocyte subsets, T cells and B cells. (B) The number of neutrophils, T cells and B cells in the spleen of young and old, male and female mice was assessed by flow cytometry. Data are presented as mean plus standard deviation of 10-15 mice in each group, and statistical significance was assessed by two-tailed Student's t test (*$p < 0.05$, **$p < 0.01$).

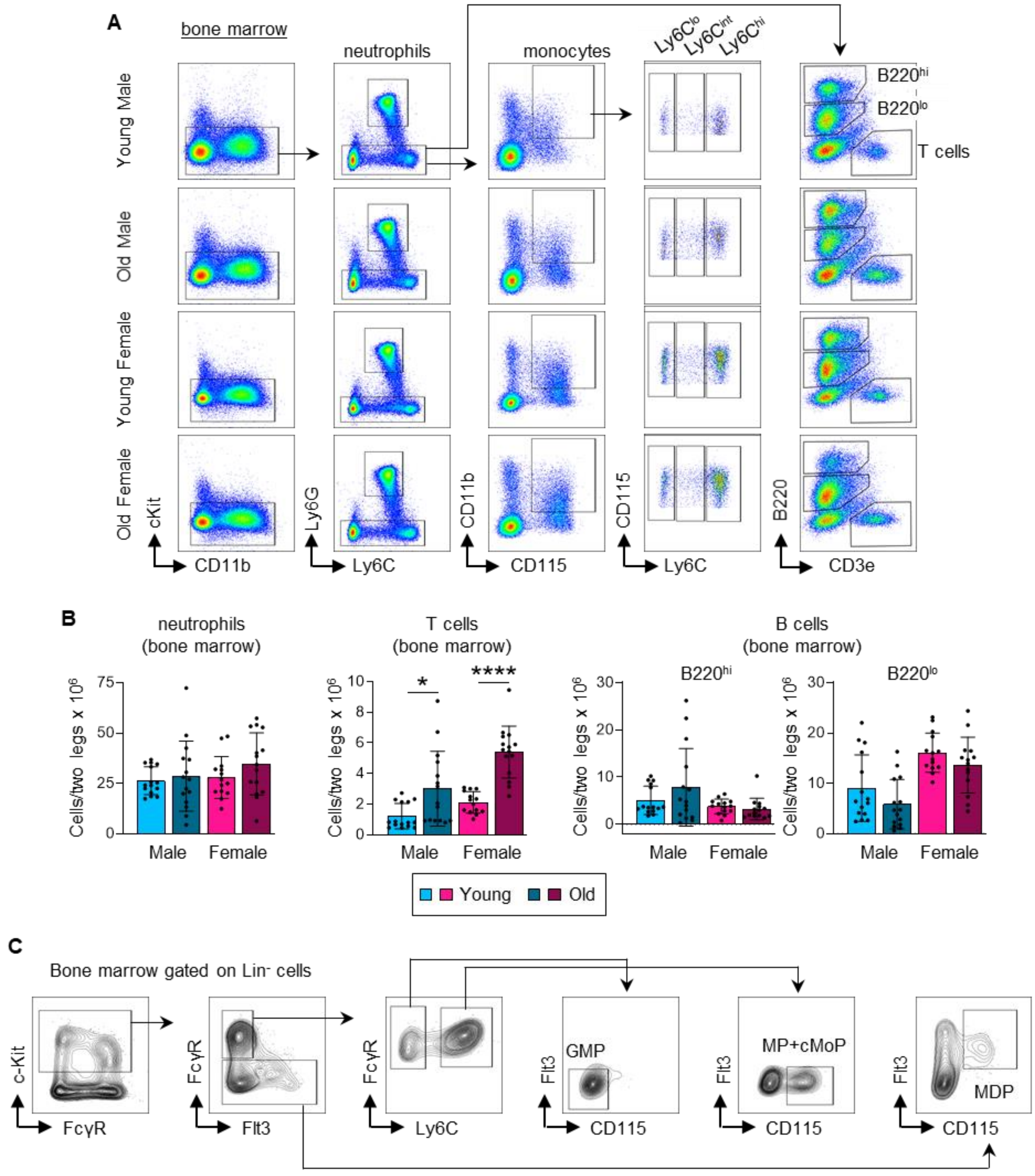

Fig. S3. Evaluation of myeloid and lymphoid cells and monocyte progenitors in the bone marrow. (A) Flow cytometry gating of bone marrow cells for the identification of neutrophils, total monocytes, monocyte subsets, T cells and B cells in young and old male and female mice. (B) The number of neutrophils, T cells and B cells (including B220^{hi} and B220^{lo} subsets) in the bone marrow of young and old, male and female mice was assessed by flow cytometry. Data are

presented as mean plus standard deviation of 10-15 mice in each group, and statistical significance was assessed by two-tailed Student's t test (*$p < 0.05$, ****$p < 0.0001$). (C) Flow cytometry gating of bone marrow Lin^- cells for the identification of granulocyte-monocyte progenitors (GMPs; Lin^- $c\text{-}Kit^+$ $Fc\gamma R^{hi}$ $Ly6C^-$ $Flt3^-$ $CD115^{lo}$ cells), monocyte-DC progenitors (MDPs; Lin^- $c\text{-}Kit^+$ $Fc\gamma R^{lo}$ $Flt3^+$ $CD115^{hi}$ cells) and monocyte-committed progenitors (MPs and cMoPs; Lin^- $c\text{-}Kit^+$ $Fc\gamma R^{hi}$ $Ly6C^+$ $Flt3^-$ $CD115^{hi}$ cells).

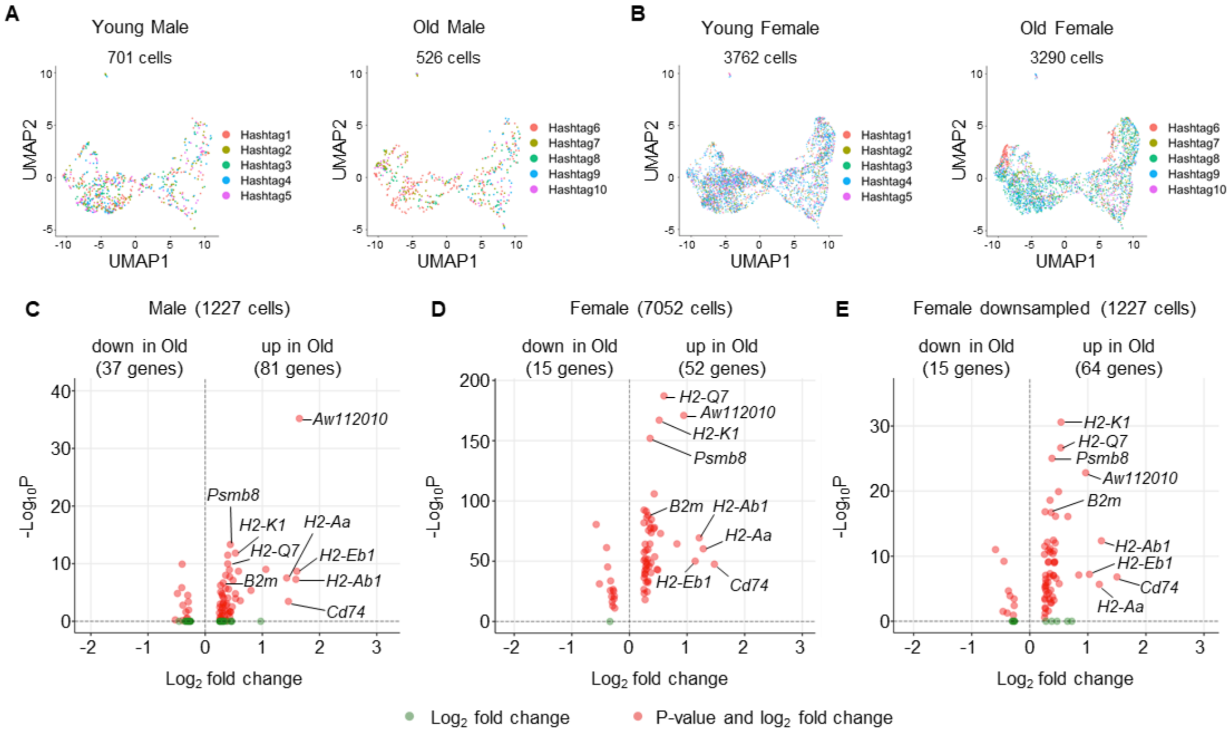

Fig. S4. scRNAseq of classical monocytes from young and old, male and female mice. (A-B) UMAP visualization of bone marrow classical monocytes showing distribution of cells from each young and old, male (A) and female (B) mouse. Hashtags 1-5 are young mice and hashtags 6-10 are old mice. (C-E) Volcano plots show DEGs between young and old bone marrow classical monocytes in male (C), female (D), and downsampled female (E) mice.

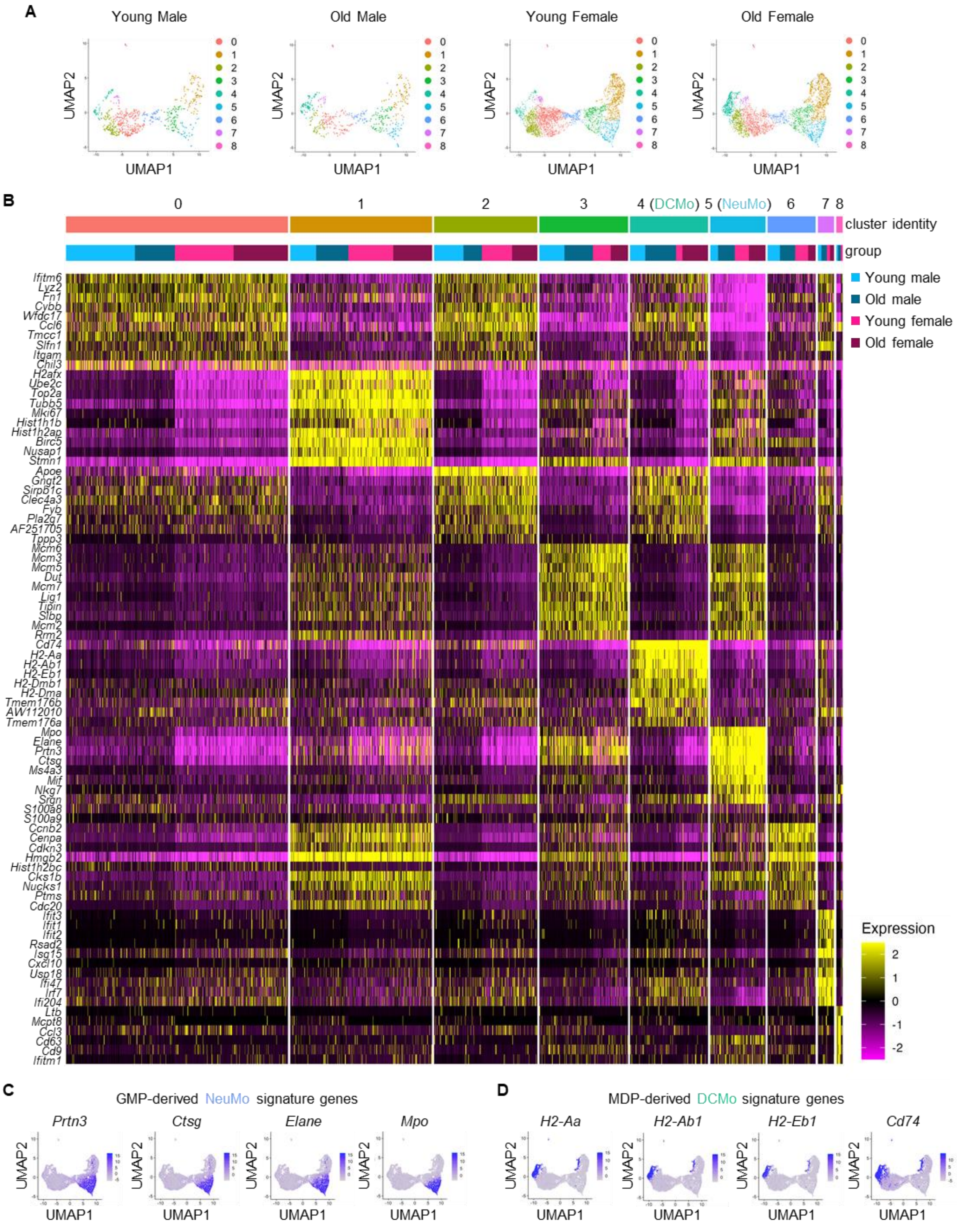

Fig. S5. Identification of classical monocyte subsets. (A) UMAP visualization of classical monocytes from young and old, male and female mice (5 mice per group) profiled by scRNAseq. (B) Top 10 signature genes for monocyte clusters defined using the combined male and female

datasets. NeuMo (cluster 5) and DCMo (cluster 4) subsets were identified using signature genes defined in our previous study (Yanez et al., 2017). (C-D) UMAP visualization showing the expression of key NeuMo (C) and DCMo (D) genes.

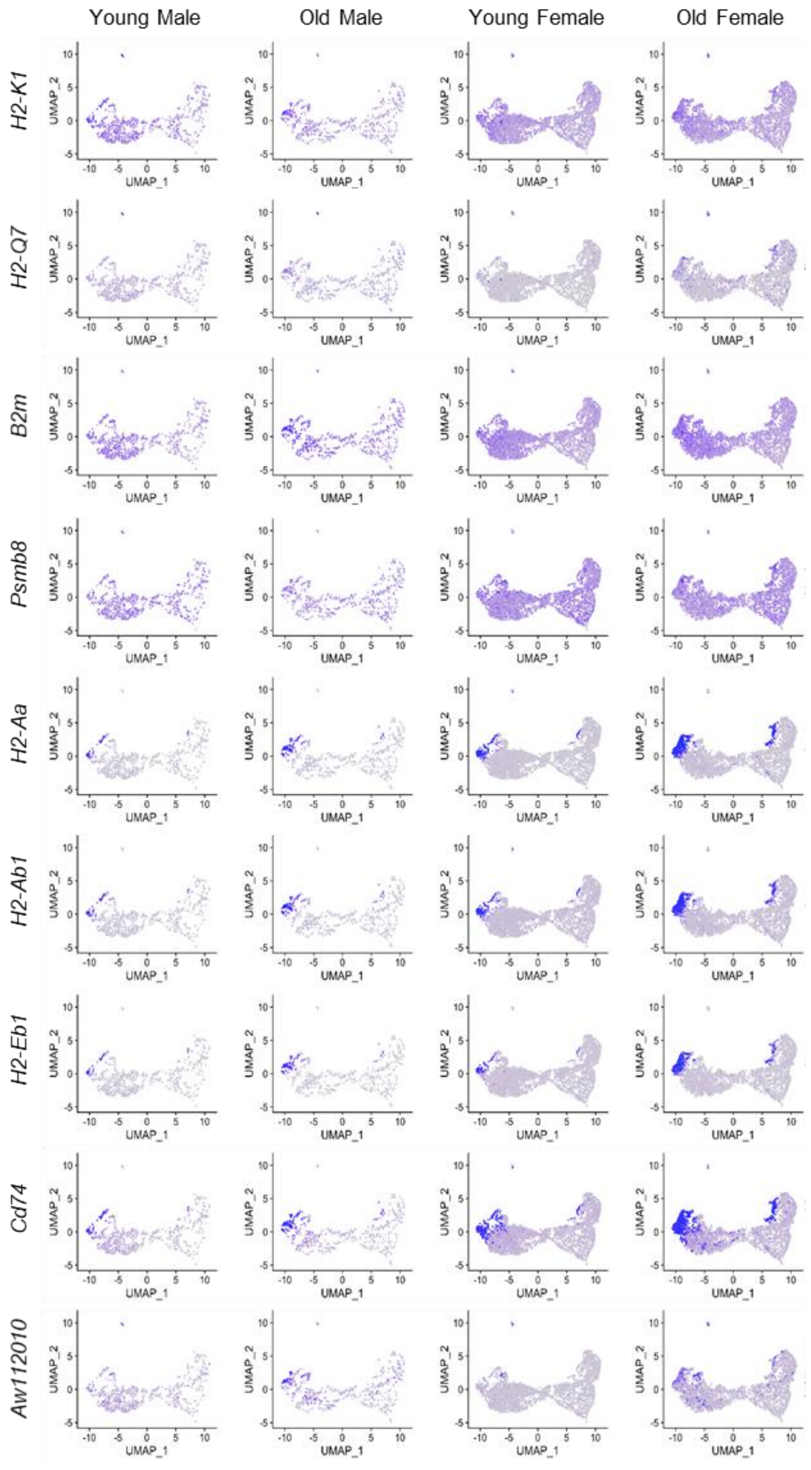

Fig. S6. Expression of aging-associated DEGs across the monocyte clusters. UMAP visualization showing the expression of MHCI and associated genes, MHCII and associated genes, and *Aw112010*.

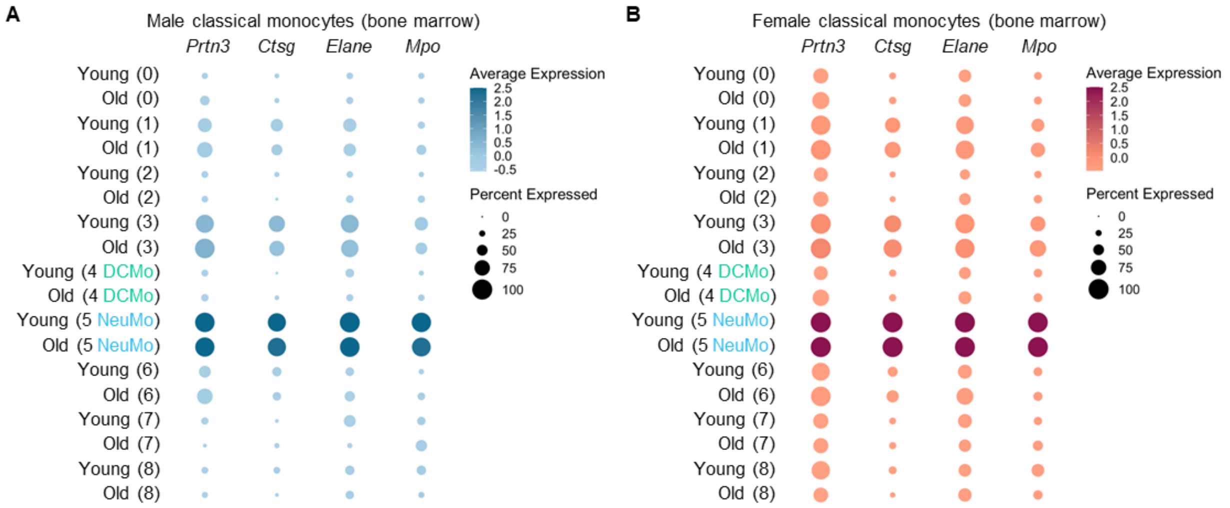

Fig. S7. Expression of NeuMo signature genes across the monocyte clusters. Dot plots show the expression of NeuMo signature genes across the monocyte clusters in young and old, male (A) and female (B) mice.

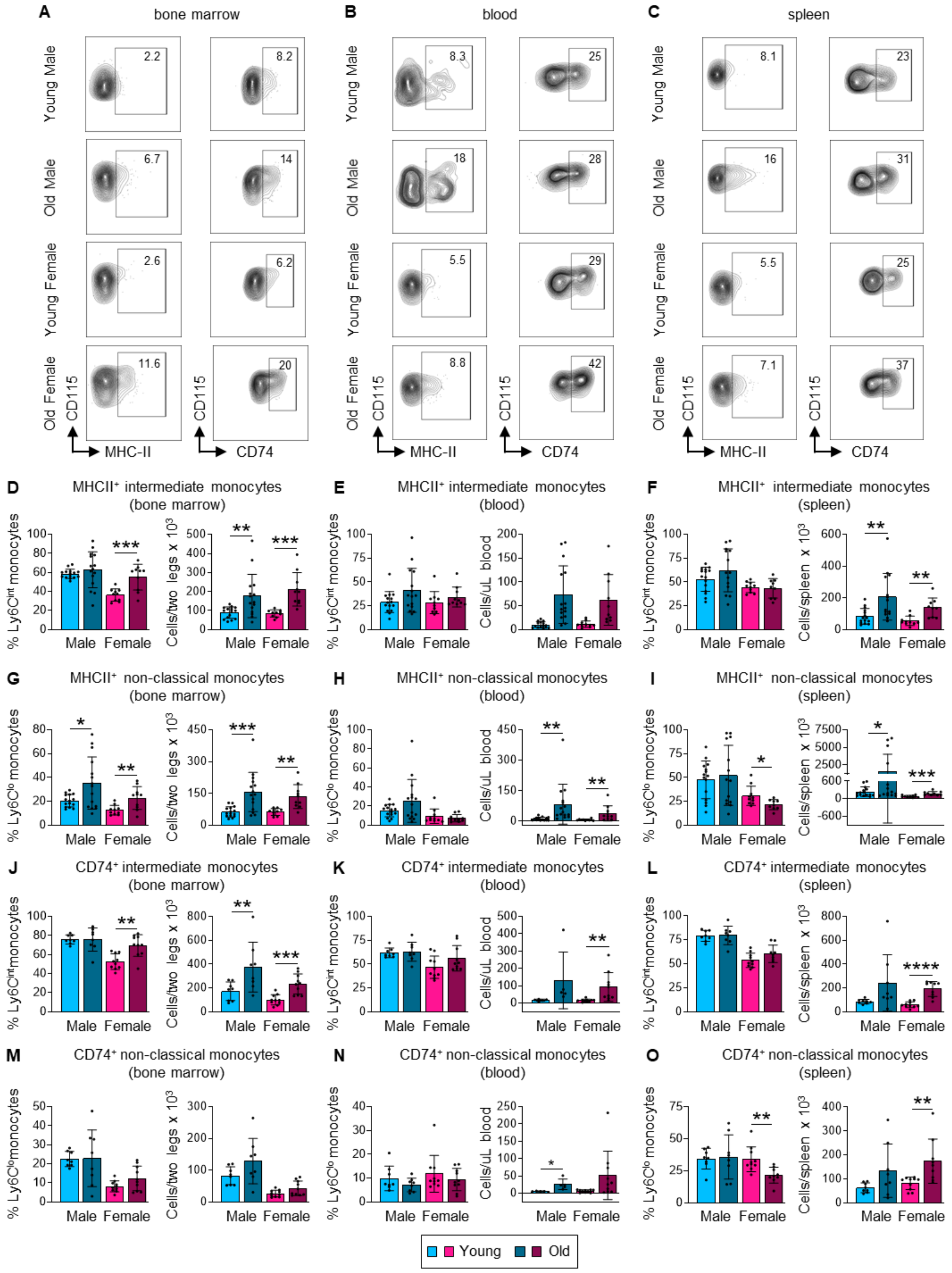

A
bone marrow
B
blood
C
spleen
Young Male
Old Male
Young Female
Old Female
CD115
MHC-II
CD74
D
MHCII⁺ intermediate monocytes (bone marrow)
E
MHCII⁺ intermediate monocytes (blood)
F
MHCII⁺ intermediate monocytes (spleen)
G
MHCII⁺ non-classical monocytes (bone marrow)
H
MHCII⁺ non-classical monocytes (blood)
I
MHCII⁺ non-classical monocytes (spleen)
J
CD74⁺ intermediate monocytes (bone marrow)
K
CD74⁺ intermediate monocytes (blood)
L
CD74⁺ intermediate monocytes (spleen)
M
CD74⁺ non-classical monocytes (bone marrow)
N
CD74⁺ non-classical monocytes (blood)
O
CD74⁺ non-classical monocytes (spleen)
Male Female
Young
Old

Fig. S8. Evaluation of MHCII and CD74 protein expression by monocytes. (A-C) Flow cytometry evaluation of surface MHCII and intracellular CD74 protein expression by classical monocytes from the bone marrow (A), blood (B) and spleen (C) of young and old, male and female mice. (D-O) Expression of surface MHCII and intracellular CD74 protein by intermediate (D-F, J-L) and non-classical (G-I, M-O) monocytes from the bone marrow (D, G, J, M), blood (E, H, K, N) and spleen (F, I, L, O) of young and old, male and female mice was assessed by flow cytometry.

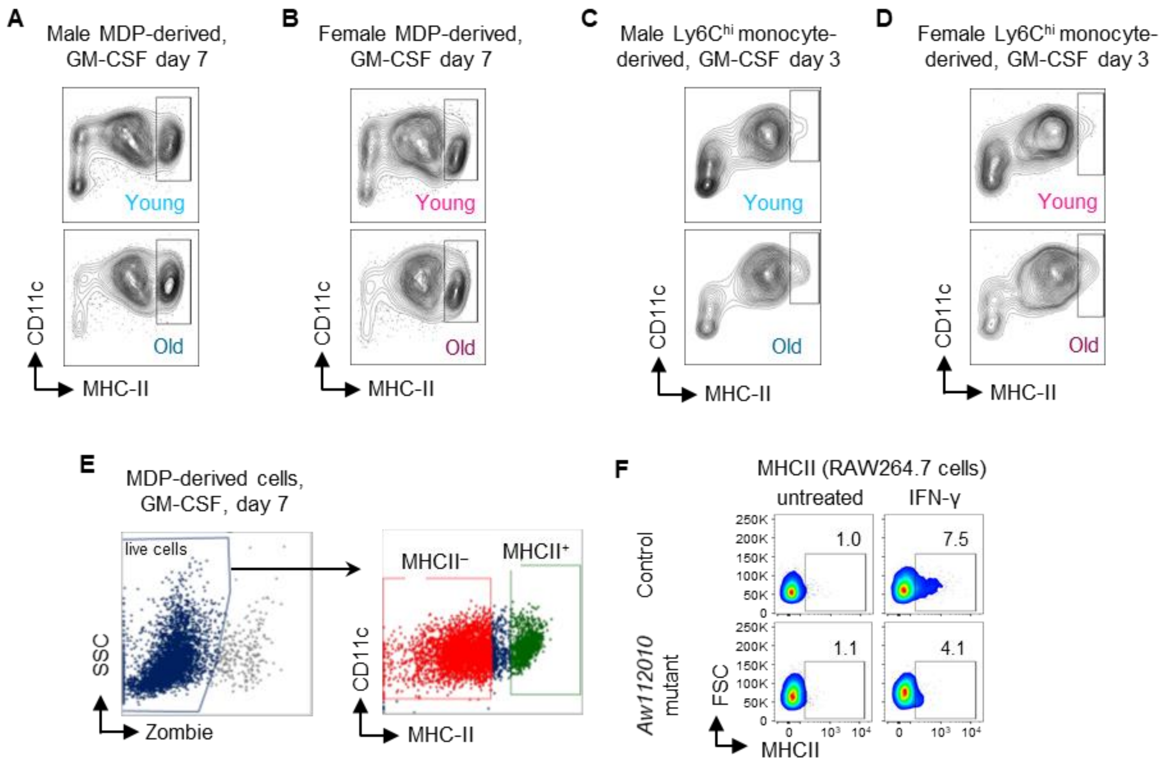

Fig. S9. Evaluation of moDC production and the role of *Aw112010*. (A-D) Representative flow cytometry plots for GM-CSF cultures of MDPs (A-B) and classical monocytes (C-D) from young and old, male and female mouse bone marrow. (E) Gating strategy for FACS sorting of $MHCII^+$ and $MHCII^-$ cells from day 7 GM-CSF cultures of bone marrow MDPs. (F) Representative flow cytometry plots for MHCII expression in control and *Aw112010* mutant RAW264.7 cells left untreated or treated with IFN-γ (10 ng/mL) for 24 hours.

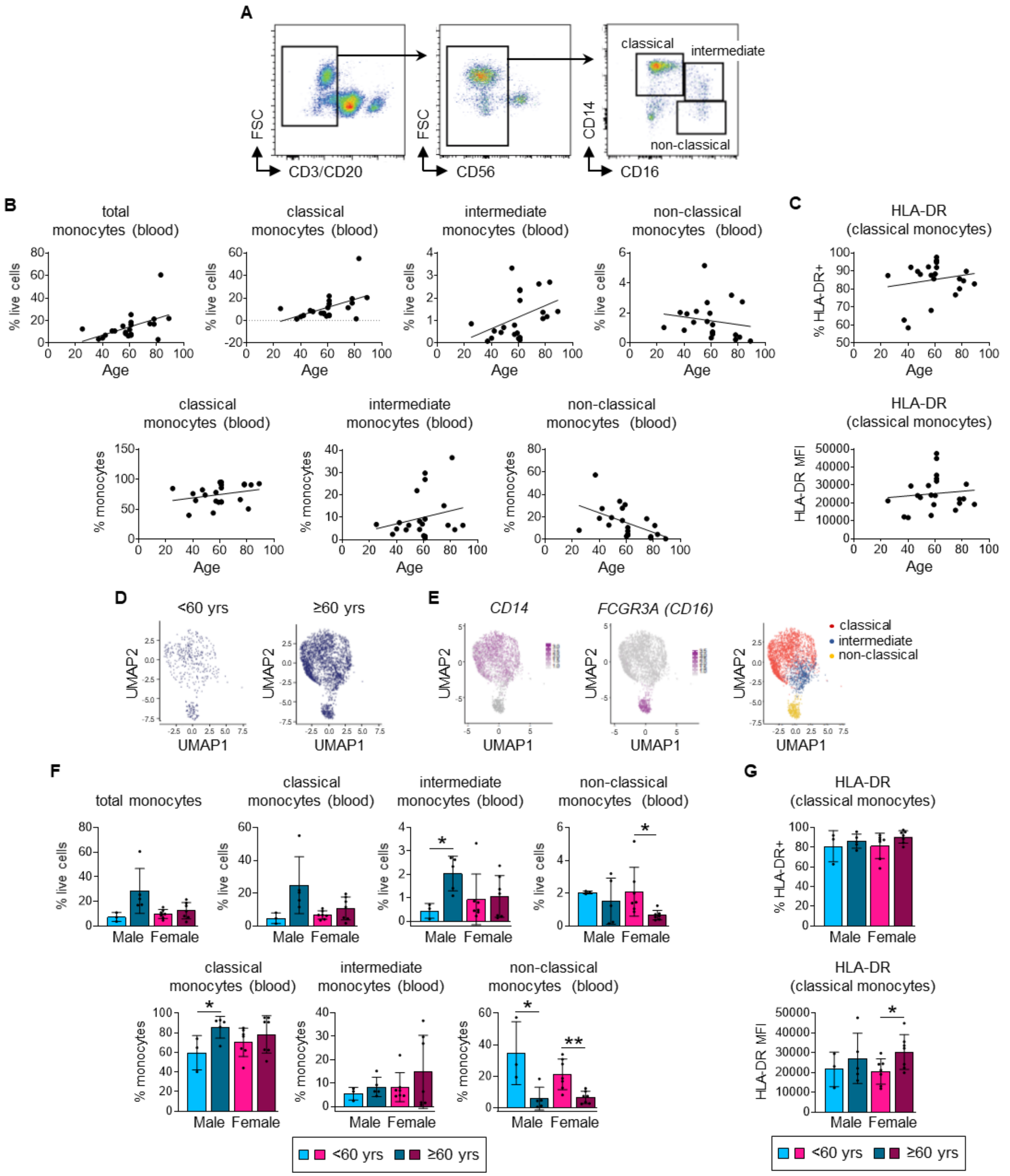

Fig. S10. Evaluation of monocyte subsets and HLA-DR expression by human classical monocytes. (A) Flow cytometry gating of human peripheral blood mononuclear cells to identify total monocytes ($CD3^{-}$ $CD20^{-}$ $CD56^{-}$ cells that are positive for CD14 and/or CD16) and classical ($CD14^{+}$ $CD16^{-}$ cells), intermediate ($CD14^{+}$ $CD16^{+}$ cells) and non-classical ($CD14^{-}$ $CD16^{+}$ cells) monocyte subsets. (B) Flow cytometry assessment of the proportion of total monocytes and

classical, intermediate, and non-classical monocyte subsets in the peripheral blood of younger (3 male and 7 female combined) and older (5 male and 7 female combined) individuals correlated with age as a continuous variable. (C) Flow cytometry assessment of HLA-DR expression by peripheral blood classical monocytes from younger and older individuals correlated with age as a continuous variable. (D-E) UMAP visualization of monocytes from younger and older individuals (4 per group, all male) assessed by scRNAseq (D), and identification of classical, intermediate, and non-classical monocytes on the basis of *CD14* and *CD16* expression (E). (F-G) Flow cytometry assessment of total monocytes and monocyte subsets (F), and classical monocyte expression of HLA-DR (G) by younger and older, male and female individuals (3 younger male, 7 younger female, 5 older male, 7 older female). Data are presented as mean plus standard deviation, and statistical significance was assessed by two-tailed Student's t test (*$p < 0.05$, **$p < 0.01$).
